# Comparison in Four Different Implant Systems of Mechanical Resistance to Maximal Stress in Prosthetic Screws—An In Vitro Study

**DOI:** 10.3390/dj8040116

**Published:** 2020-10-09

**Authors:** Pedro Barreiros, Luís Neves, Carlos Aroso, José M. Mendes, António Sérgio Silva

**Affiliations:** 1 Department of Oral Rehabilitation, Instituto Universitário de Ciências da Saúde Rua Central da Gandra 1317, 4585-116 Gandra, Portugal; a18596@alunos.cespu.pt (P.B.); a17615@alunos.cespu.pt (L.N.); 2 Dental Science Department, Instituto de Investigação e Formação Avançada em Ciências e Tecnologias da Saúde (IINFACTS), Rua Central da Gandra 1317, 4585-116 Gandra, Portugal; carlos.ribeiro@iucs.cespu.pt (C.A.); jose.mendes@iucs.cespu.pt (J.M.M.)

**Keywords:** screw, abutment, implant, fracture, implantology

## Abstract

Micromovements of the implant–abutment connection influence peri-implant bone preservation. This study evaluates and quantifies the maximal torque after a cycle of implant prosthetic screws tightening using original components. A total of 40 samples were tested: Megagen^®^—Daegu, South Korea; Dentium^®^—Gangnam-Gu, Seoul, Korea; BIOMET 3i^®^—West Palm Beach, FL, USA and BTI^®^—Álava, Spain. Screws from each manufacturer were subjected to maximal stress force until they fractured. The fracture points were recorded and compared among all samples. To compare the mean values of fracture torques, the reference values associated with each brand and the sample results were used in *t*-tests. ANOVA (analysis of variance) was used to compare the maximal resistance limit between brands, complemented with Tukey’s multiple-comparison test. The maximal considered level of significance was 5%. The average fracture force for the brands was 40.07 Ncm for Megagen^®^, 53.39 Ncm for Dentium^®^, 39.74 Ncm for Biomet 3i^®^, and 68.84 Ncm for BTI^®^. BTI^®^ screws showed the most resistance to fracture. According to the protocol that was applied, the implant–abutment connection demonstrated good resistance and a precise fit between these interfaces; therefore, in some cases, the presented values showed a lack of quality control and low fracture resistance.

## 1. Introduction

The loss of natural teeth has been a problem for human beings throughout their existence. In primitive societies, most tooth losses were due to trauma, whereas at present, tooth loss is associated with diseases of the oral cavity, the most prevalent being dental caries and periodontal disease. The use of dental implants is of great importance in restoring an edentulous patient’s masticatory, aesthetic, and phonetic functions, contributing to improvements in their oral health and quality of life [1,2].

The success of implant-supported rehabilitation depends on the direct interaction between bone and implant surface without the interposition of connective tissue or signs and symptoms of infection or inflammation [3]. In addition, the hermeticity and durability of all prosthetic components are associated with this same rehabilitation [4]. One of the most prevalent problems associated with dental implants is the loosening and fracture of the implant–abutment connection screws. Such loosening is an indicator of inadequate biomechanical design and/or occlusal overload [5,6].

This complication has been established as comprising 6% of prosthetic complications [7,8,9]. The cumulative incidence of complications related to the type of connection is from 5.8% to 12.7% in 5 years [10,11], and only 35% of screws remain stable in the first 3 years [12,13]. Moreover, 26% of screw-retained prostheses and 43% of the screws loosen in the first year of function [14,15,16]. Clinically, screw loosening is more important in cemented prostheses because it is more difficult to remove the crown to solve the problem [17,18,19].

Screw loosening is more frequent in external connections (prevalence of 38%) than in internal connections and in single implants, although it also occurs in multiple prostheses. However, in the case of multiple prostheses, the perception of screw loosening by the professional and the patient is much more complicated and can lead to the appearance of fractures or bone loss around the implants that still have the screws tightened due to overload, in addition to the possibility that the screws of these implants also loosen [20,21,22].

On the other hand, screw loosening can lead to the appearance of biological problems at the implant–abutment interface, since it allows bacteria to leak through the interface. This bacterial leakage, together with the mechanical problems derived from screw loosening, can lead to the loss of peri-implant bone, as well as the appearance of infiltrate of inflammatory cells. Furthermore, with the presence of inflammatory infiltrate at the level of the alveolar bone, the possibility of mechanical failure and fatigue and the risk of screw loosening increase. Several studies have linked the presence of periodontal pathogens in the peri-implant sulcus with the appearance of damage to the adjacent hard and soft tissues, leading to implant failure [23,24]. However, this rehabilitation procedure, in a prosthetic point of view, has complications: screw fractures can occur under functional cyclic load [5,6]. The abutment screw may be overloaded and fracture, leaving the abutment and coronal screw fragment within the final abutment/crown and the apical fragment in the support itself [6,7].

Occlusal load is a force of multidirectional and variable magnitude [5,8,9]. Although an integrated implant transmits the load to the surrounding bone, the load is transmitted through the interface and its retaining screw. There is slight oscillation of the interface during functional loads [9,10,11]. The pillar/interface screw is subject to traction and flexion movements that can induce fatigue fractures, which are the main cause of screw fractures [12,13].

Screw fractures are reduced when using gold alloy screws or passive connection structures without any mechanical stress on the fit [14,15]. It is important to understand the risks that are inherent in the use of original overloaded screws and the behaviors of different brands, in addition to establishing the confidence interval between the information provided by the manufacturer and the observed value in an investigation [16]. The fracture and loosening of the screws that connect the prosthetic component to the implant are the main causes of failure in implant-supported rehabilitation [17,18,19].

The screw is tightened by applying force torque. The applied torque develops a force on the screw that is called the preload. As the screw is tightened, it stretches, producing tension. The elastic recovery of the screw brings the two components that are tightened together, creating a clamping force. In contrast to the clamping force, there is a separating force between the two parts that are joined by the screw. Screw loosening occurs when the separation force is greater than the clamping force. Excessive force causes the screw turns to slip, with a loss of preload [19,20].

The preload is determined by factors such as applied torque; the metal alloy, design, and surface of the screw; and the prosthetic parts. The preload is proportional to the applied torque. In other words, insufficient torque allows for a separation between prosthetic part and implant, and too much torque may grind the screw turns. The ideal torque is 75% of the torque that is required to break the screw [14,15].

One variable is the amount of torque that can be applied to the screw and how it is applied: manually via a mechanical device, or digitally. On average, manually applied force varies between 10 and 15 Ncm^2^. In addition, the torque indicated by the manufacturer is on average between 20 and 35 Ncm^2^. There are intraoral separation forces that include off-axis occlusal forces, excursive laterality movements, interproximal contact points, protrusive movements, parafunctional forces, and structures without passive laying [21,22].

As soon as masticating forces exceed the preload, the seating and connection become unstable, with vibration and micromovements leading to the loosening of the screw, i.e., a loss of its function and a mechanical failure [21,22]. In addition, since the surface of the screw is not completely smooth, which is known as microroughness, contact surfaces do not completely come into contact. Settling occurs when irregular points flatten under a load, as they are the only contact surfaces when the initial tightening torque is applied. It has been reported that 2% to 10% of the preload is lost as a result of relaxation forces, which is known as the settling effect. As a result, the torque required to remove the screw is less than the torque initially used to place the screw. To reduce this phenomenon, the screw must be tightened 10 min after initial torque application [23,24].

The purpose of this study was to evaluate and quantify the maximal torque after a cycle of tightening of an implant prosthetic screw using the original components.

## 2. Materials and Methods

### 2.1. Study Characterization

The study was a descriptive and inferential experiment.

### 2.2. Sample Characteristics

A total of 40 prosthetic screws from the following brands were tested: Megagen^®^—Daegu, South Korea; Dentium^®^—Gangnam-gu, Seoul, Korea; BIOMET 3i^®^—West Palm Beach, FL, USA and BTI^®^—Álava, Spain. This study was performed by using 10 laboratory analogs of each brand and 10 prosthetic abutments, where 10 original screws from each manufacturer were tested. In each prosthetic screw, a brand-new screwdriver and torque wrench were used.

**Figure 1:**
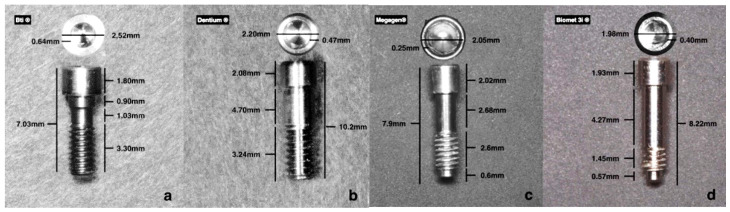
Screw caractheristics and dimensions. (**a**) Bti^®^; (**b**) Dentium^®^; (**c**) Megagen^®^; (**d**) Biomet 3i^®^.

The prosthetic screws characteristics are described in Figure 1, with length, diameter of internal connection, and size of each region (head, neck, and threads).

### 2.3. Inclusion and Exclusion Criteria

Inclusion: prosthetic screws, direct implant connection, titanium alloys of the same grade.

Exclusion: prosthetic screws from nonoriginal brands.

### 2.4. Data Collection

A standard laboratory protocol was established and applied at the Institute for Research and Advanced Training in Health Sciences and Technologies (IINFACTS-CESPU) to test all selected samples.

**Figure 2:**
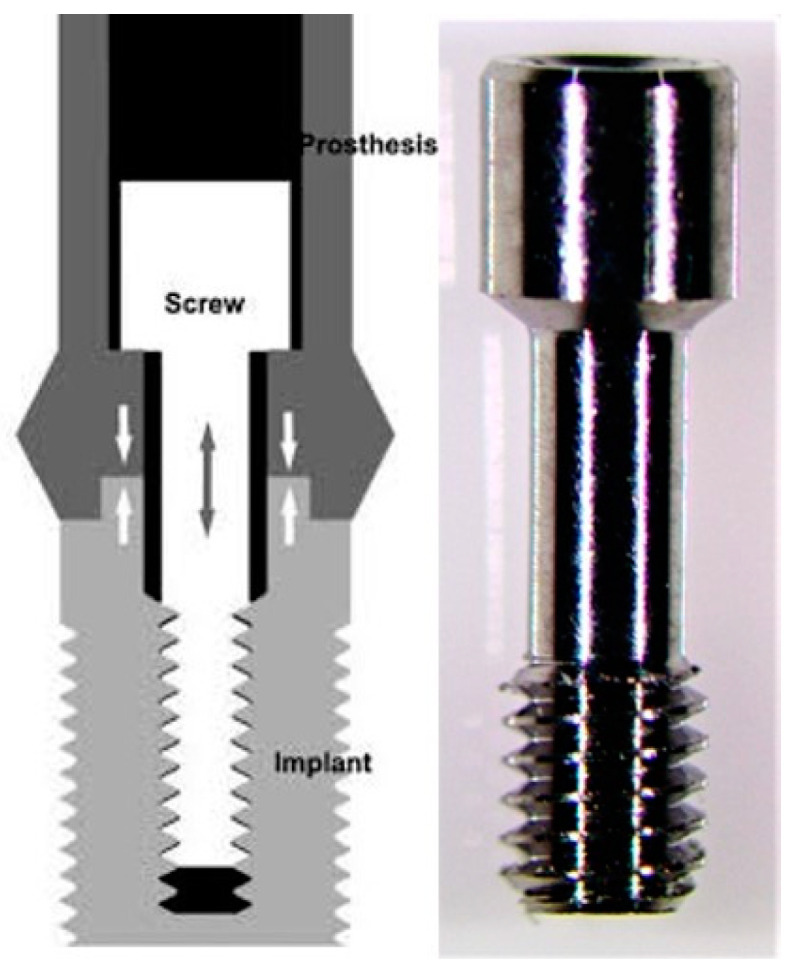
Example of prosthetic screw—Dentium^®^.

**Figure 3:**
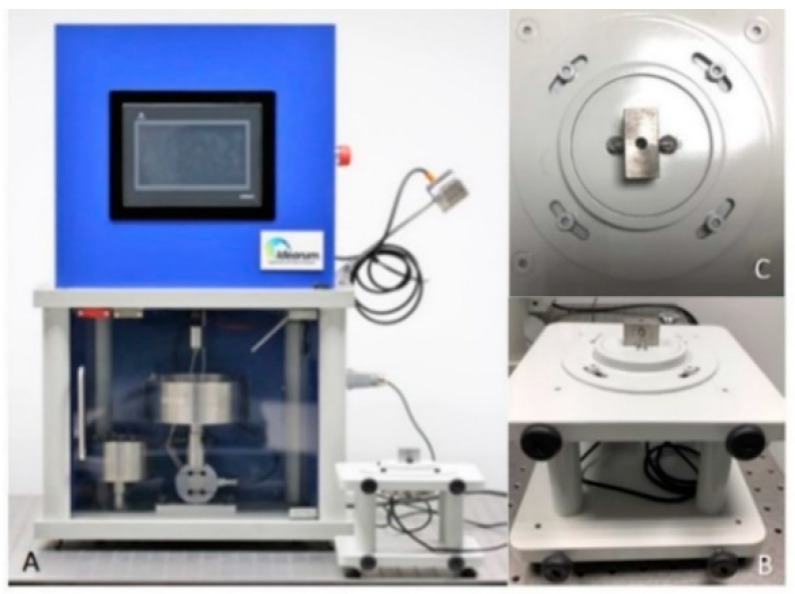
(**A**) CS-Dental Testing Machine^®^; (**B**) load cell; (**C**) load cell (superior view) with retention area to prosthetic abutments.

(1) All prosthetic screws from the manufacturers were labeled with serial number and control date: 10 Megagen^®^ prosthetic screws; 10 Dentium^®^ prosthetic screws; 10 BIOMET 3i^®^ prosthetic screws and 10 BTI^®^ prosthetic screws.
(2) The presence of anomalies and defects was assessed with a stereoscope (Olympus^®^ SZ61—Tokyo, Japan), and a 90× magnifier was used to evaluate any changes in the surfaces.
(3) The prosthetic abutment was coupled to the implant analog (of the corresponding brands) with the prosthetic screw (Figure 2) with the respective manual key. The prosthetic screw was tightened by hand until there were no gaps between the two parts.
  A brand-new manual screwdriver was used every time a prosthetic screw was tested.
(4) The two parts were placed in a load cell (Figure 3B,C), connected to the CS-Dental Testing Machine^®^ (Figure 3A), and stabilized. CS^®^ Dental Testing Machine is a fatigue test device built in agreement with 2006/42/CE safety of machines and the norms EN 12100-1/2, EN 954-1, EN 1037, EN 61310-1/2, EN 60204-1, EN ISSO 14121-1, and EN ISSO 13850.
  The screws to be tested were subjected to a torsional force with a torque wrench until fracture occurred. A brand-new torque wrench was also used every time a prosthetic screw was tested.
(5) The fracture points were automatically recorded in the machine and compared between all samples.
(6) The CS-Dental Testing Machine^®^ (Barcelona, Spain) Excel file was stored on a computer for further graphical and value analyses.
(7) The fractured parts were analyzed under an optical microscope to observe microscopic fracture characteristics.

### 2.5. Statistical Analysis

Data analysis was carried out in SPSS, version 24. First, exploratory data analysis was performed, which detected two outliers, one in Dentium^®^ and another in Biomet 3i^®^. As these extreme values were not explained by the methodological process, an imputation process for missing values was carried out. Those extreme values were replaced by the means of the remaining nine values obtained in the tests, 53.39 and 39.74 Ncm, respectively.

In descriptive analysis, the means and standard deviations for the fracture torques, expressed in Ncm, were calculated after symmetric distribution was confirmed by observing the histograms.

Normal distribution was assessed using the Shapiro–Wilk test, used for n < 50, confirming the necessity of parametric tests. The four brands’ homogeneous distribution was evaluated with the Levene test, confirming this assumption.

To compare the mean values of fracture torques, the reference values associated with each brand and the sample results were used in the *t*-test. The reference values for each brand were 35 Ncm for BTI^®^ and Megagen^®^, 30 Ncm for Dentium, and 20 Ncm for BIOMET 3i^®^.

ANOVA was used to compare the maximal resistance limit between brands. This test was complemented with Tukey’s multiple-comparison test.

The maximal considered level of significance was 5%.

## 3. Results

A total of 40 samples were tested from the following brands: Megagen^®^, Dentium^®^, BIOMET 3i^®^, and BTI^®^ (Table 1).

When comparing the average fracture points of the four brands, we obtained the following values (Table 2). Bti^®^ had the highest mean value followed by Dentium^®^, Megagen^®^, and BIOMET 3i^®^, respectably.

**Figure 4:**
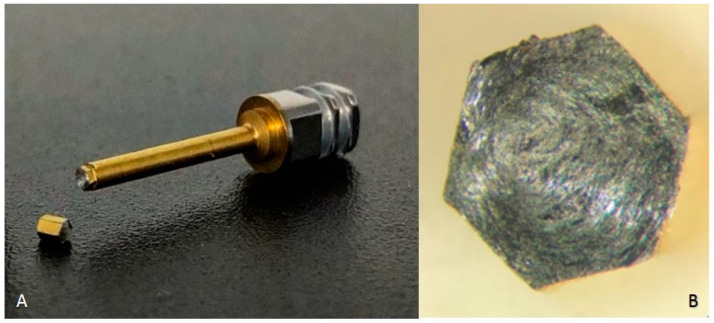
(**A**) Fracture point in detail—BTI^®^ prosthetic key; (**B**) directional force of torsion (fracture pattern).

There were three fractures of the prosthetic tightening wrenches from the BTI^®^ brand. The fractures of the three prosthetic keys occurred at 46, 40, and 43 Ncm (Figure 4A). When observing these, the same fatigue pattern, which was torsion until the maximal point of elasticity of the material, was verified (Figure 4B). In this case, for the continuity of the work, it was necessary to use a compatible key that was made of a more resistant titanium alloy.

**Figure 5:**
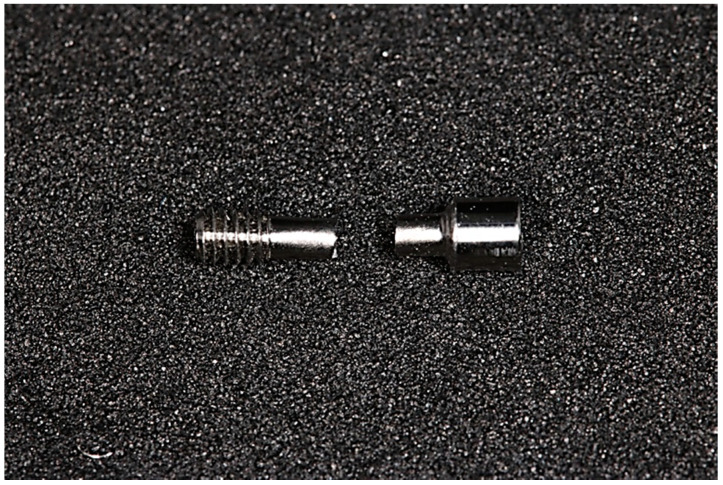
Fracture point of all studied samples.

The deformation of the prosthetic screw always occurred in the head area, which was a transitional region from the head to the screw thread (Figure 5).

**Figure 6:**
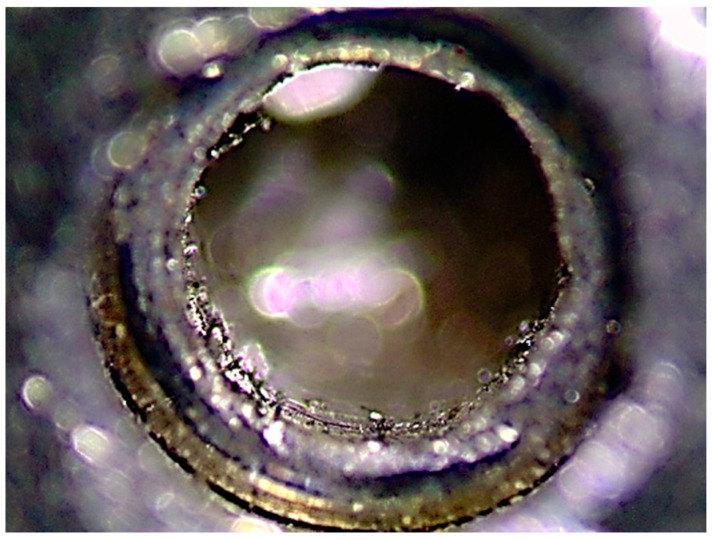
Representative grinding of prosthetic screw fit.

When the fracture-torque variable was analyzed, the screw brand with the maximal torque was BTI^®^, with a maximal torque of 78.30 Ncm. The minimal torque, curiously, was 13.18 Ncm in Dentium^®^. This finding led us to believe that there may have been an error in the machining of the prosthetic screw thread that resulted in the grinding of the fit of the prosthetic screw (Figure 6), leading to the conclusion that the prosthetic wrench could not perform its function.

**Figure 7:**
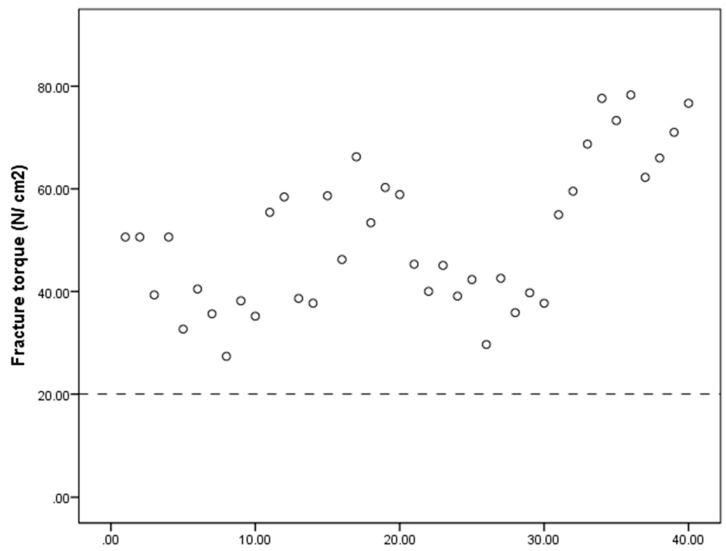
Distribution of maximal fracture-resistance torque limit of all analyzed samples.

Figure 7 shows the fracture torque measurement distribution. All measurements were greater than 20 Ncm.

Table 3 presents the *t*-test results comparing the mean measured values of fracture-resistance torque with the reference values presented by each of the brands. In the Dentium^®^, BIOMET 3i^®^, and BTI^®^ brands, the mean of the maximal fracture-resistance torque limit was significantly higher (*p* < 0.001) than the maximal resistance limit referenced by the manufacturers. Megagen^®^ did not produce statistically significant results (Table 3).

To assess the maximal loads proposed by manufacturer calibration, the number of cases below the limits proposed by the brand were calculated. Values below the reference limit were only found for Megagen^®^ samples (n = 2; 20%) (Table 4).

When comparing the mean values of fracture torque between the brands, statistically significant differences were found: F_(3.36)_ = 31.20 (*p* < 0.001), evidenced by Tukey’s multiple-comparison test, with differences in BTI^®^, which produced the highest mean value; BIOMET 3i^®^, which produced the lowest mean value when compared with Dentium^®^, BTI^®^, and Megagen^®^; and Megagen^®^, compared with Dentium^®^, had the lowest mean value (Table 3).

The mean fracture torque values observed for the four brands were 40.07 Ncm for Megagen^®^, 53.39 Ncm for Dentium^®^, 39.74 Ncm for BIOMET 3i^®^, and 68.84 Ncm for BTI^®^. The screws that showed more resistance to fracture were those from BTI^®^.

The BTI^®^ brand had a much superior result and was the only screw that presented an extra characteristic on the part of the manufacturer. The screws of this brand are surface-treated with tungsten carbide, which apparently reduces the friction coefficient and improves the slip, giving the screws similar properties to those of gold in terms of pretension and resistance to fatigue, i.e., a greater preload for the same tightening pair and reduced screw loosening.

## 4. Discussion

When using dental implants with screws for prosthetic rehabilitation, the correct amount of torque on the screw is essential for the ideal preload of the implant union, which is the prosthetic abutment.

The loosening or fracture of prosthetic screws is related to the mismatch of the implant–prosthetic abutment and the presence of a gap between the implant connection and the prosthetic abutment, which may cause unfavorable stresses on the connection components, implant, and bone. Screw loosening or fractures are common complications of implant-supported rehabilitation. The gap between implant and prosthetic abutment may have a significant influence on these complications [25]. Many authors have reported that screw loosening is one of the most common prosthetic complications in rehabilitation with implants, which may be related to the tightening technique or insufficient torque. Some authors have reported that the higher the torque, the greater the preload and the lower the probability of the screw loosening and consequently disinserting from the prosthetic abutment [26,27].

The screw in a screw connection will only become loosened if external forces that induce the separation of the parts are greater than the force that holds them together. Therefore, the separation forces of the connection must not be eliminated but instead kept below the limit of the joining forces [24,27]. Most authors and brands on the market advocate for a maximal tightening torque of 20−35 Ncm [28,29,30]. The manufacturers of the studied brands recommend different reference values: 35 Ncm for BTI^®^ and Megagen^®^, 30 Ncm for Dentium^®^, and 20 Ncm for BIOMET 3i^®^, which are in line with the comparative studies [29,30].

Therefore, the two main factors that are involved in keeping implant screws tight are maximization of the locking force and minimization of the separation forces in the connection. The strength of the connection is more affected by the locking force than by the strength of the screws. The bonding force is proportional to the tightening torque. However, to increase bond strength and consequently preload torque, greater resistance values of the prosthetic screws are needed. Low torque values allow for separation of the connection and result in screw fatigue or loosening. Excessive torque can cause the screw to fail or deform the screw threads, which ultimately leads to screw fracture.

i. The retightening of screws 10 min after application of the initial torque should be routinely performed. This counteracts the memory effect of the prosthetic screw [23,24].
ii. Increasing the screw torque above 30 Ncm could be beneficial for implant–abutment stability and reducing screw loosening [23].

The strength of the screw material has a significant influence on the preload [23]. The manufacturers of screw connections recommend tightening to 75% to 80% of the material’s elastic limit as a reserve to avoid permanent deformations. This was verified in this study, where the average resistance of the screws to fracture was higher than the limit stipulated by the manufacturer.

The average fracture-torque values of prosthetic screws from the different brands were 39.74 Ncm for BIOMET 3i^®^, 40.07 Ncm for Megagen^®^, 53.39 Ncm for Dentium^®^, and 68.84 Ncm for BTI^®^, with increasing resistance.

It seems logical that the stronger the screw is, the greater the preload that can be achieved. This is only partially true, since after a certain amount of tightening the screw, the friction between the implant threads and screw becomes so great that the hexagon of the screw head dusts or the installation key breaks.

Although one aspect that should be considered for reducing screw loosening is the application of higher torque values—in the designs of the screws studied here, torque values of 40 Ncm could be applied without plastic deformation—it turns out that there are screws that fracture below the values described in the present study [23,28]. Therefore, the use of higher torque values, despite increasing preload and providing an increase in the resistance to separation of the connection and greater stability to the screw [31], could also induce irreversible deformation and fracture of the prosthetic screws in certain batches of screws.

The Megagen^®^ screw did not have statistically significant results when comparing the mean value of torque resistance with the reference value approved by the manufacturer, which may imply a key point that this reference value must not be exceeded. Two screws from this brand, when evaluated for the calibration of the maximal stipulated torque, gave values below the reference limit, which in the sample size was significant, as it represented 20% of the total sample. This could be an indicator of either deficient screw strength and resistance, or some quality-control error of the material.

Abutment design can determine the point at which a prosthetic screw fractures in the case of excessive force on the suprastructure–implant complex, which is an important consideration in terms of the size of the screw in question. It is preferable that this fracture point be in an area with favorable access and visibility, such as the head area of the prosthetic screw, where the fragment can be more easily removed and grasped [25,26]; this pattern was observed in all screws.

Some errors in the machining of the prosthetic screw threads were verified and led to either the grinding of the prosthetic-screw socket and/or early fractures (in the preload), leading to the belief that some screws are of inferior quality, even within the same brand. According to the literature [23,24], it is advisable to retighten the screw at some time after the initial tightening to the torque that is recommended by the manufacturer. This was supported by the present results since, by canceling the “settling effect”, we could also reduce the risk of screw loosening and/or fracturing and avoid excessive tightening, which could lead to the grinding of the screw head or even to similar fractures, as verified in some screws and described by several authors [23,24].

Another factor to be considered is that the brand that had the greatest resistance was BTI^®^, which may have been due to the surface treatment applied to the screws. This was in line with existing studies [32,33,34] showing that screws in gold or with surface treatments with characteristics similar to gold have lower loosening and fracture rates [34].

Finally, fractures were observed of the three original prosthetic keys in the BTI^®^ brand, and this phenomenon occurred at a torque that was slightly higher than the manufacturer’s maximal limit. A key fatigue pattern was established, and the resilience of materials, such as the protection and conservation of prosthetic screws, could be a study focus. Although this was not examined in the present study, it opens a line of investigation for the future [35].

The type of connection between implant and abutment also plays a significant role in the long-term effect of dental implant treatment (conical internal hex—Megagen^®^ and Dentium^®^, friction-Fit hex—Biomet 3i^®^, and four-lobed internal—Bti^®^). Internal connection geometry influences the degree of abutment movement, requiring a significantly greater pull force to separate abutment from the implant, leading to conservation of prosthetic screw and less prosthetic complications [36], so this should be considered in further studies about long-term implant-rehabilitation success.

The limitations of this study are precisely the comparison between different internal connections if they influence the fracture pattern or result in a better screw-abutment performance. It is also possible that with different angulation forces (masticatory cycles), the results could be different.

## 5. Conclusions

Based on the laboratory experiments of the present study, the applied methodology, and the obtained results, we conclude the following:

(1) The greatest resistance, evidenced by the highest load torque before screw fracture, was achieved by BTI^®^;
(2) The maximal average fatigue loads were within the parameters defined by the manufacturers. However, there were prosthetic screws that did not reflect this reference value; and
(3) There seemed to be a correlation between better results for screws and surface treatment.

Manufacturers’ prosthetic screws torque should be respected and a higher preload to torque (more 5–10 Ncm) should be applied to prevent the settling effect.

## Figures and Tables

**Table 1:** Screw-fracture points for all brands.

Sample	Fracture Point (Ncm)			
	Megagen^®^	Dentium^®^	BIOMET 3i^®^	BTI^®^
1	50.60	55.43	45.31	54.95
2	50.60	58.42	40.02	59.54
3	39.33	38.64	45.08	68.72
4	50.60	37.72	39.10	77.63
5	32.66	58.65	42.32	73.31
6	40.48	46.23	29.67	78.30
7	35.65	66.24	42.57	62.24
8	27.36	13.18	35.88	66.01
9	38.18	60.26	22.54	71.01
10	35.19	58.89	37.71	76.68

**Table 2:** Mean values for each brand.

	Megagen^®^	Dentium^®^	BIOMET 3i^®^	BTI^®^
Mean (Ncm)	40.07	53.39	39.74	68.84

**Table 3:** Comparison of mean fracture torque values according to each brand’s reference values.

Brand	M	DP	Reference Value	*t*-Test (*p*-Value)
Megagen^®^ (n = 10)	40.07	8.14	35 N/cm^2^	t_(9)_ = 1.97 (*p* = 0.081)
Dentium^®^ (n = 10)	53.39	9.51	30 N/cm^2^	t_(9)_ = 7.78 (*p* < 0.001)
BIOMET 3i^®^ (n = 10)	39.74	4.66	20 N/cm^2^	t_(9)_ = 9.58 (*p* < 0.001)
BTI^®^ (n =10)	68.84	8.05	35 N/cm^2^	t_(9)_ = 19.17 (*p* < 0.001)

**Table 4:** Proportion of cases below reference limit.

Brand	n	%
Megagen^®^ (n = 10)	2	20.0%
Dentium^®^ (n = 10)	0	0%
BIOMET 3i^®^ (n = 10)	0	0%
BTI^®^ (n = 10)	0	0%
